# Mario Vrandecic, Cardiovascular Surgeon, University Professor, Researcher, Entrepreneur, Businessman, and Leader way ahead of his time

**DOI:** 10.21470/1678-9741-2020-0610

**Published:** 2020

**Authors:** Leonardo Ferber Drumond

**Affiliations:** 1 Cardiovascular Surgeon, Specialist by the Sociedade Brasileira de Cirurgia Cardiovascular, Brazil.; 2 Biocor medical staff member since 1994, Belo Horizonte, MG, Brazil.

**Mario Vrandecic:**
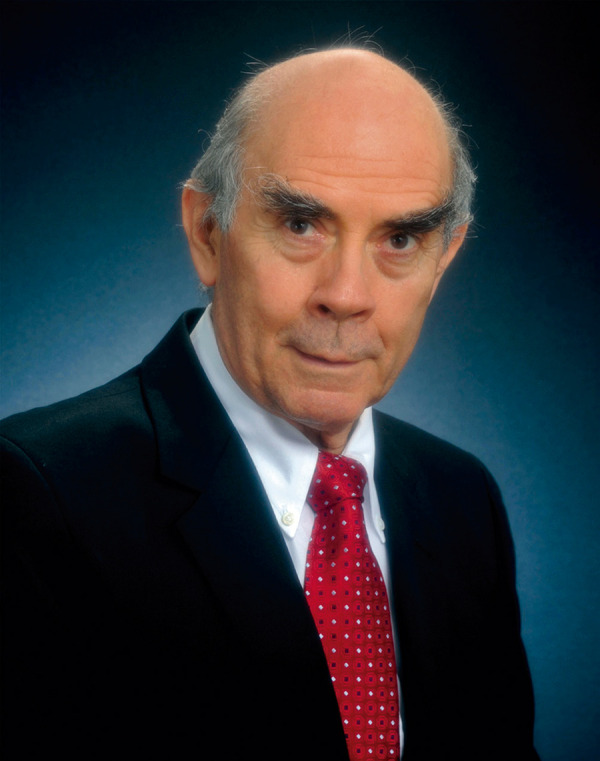

Mario Vrandecic was born in Cochabamba, Bolivia on December 8th, 1942. His mother, Alcira Peredo, was Bolivian and his father, Juan Vrandecic, Croatian.

Since childhood, learning from his parents' example, he knew he could do more for the betterment of society. He always had a vision that education would be the key to accomplishing his own personal and humanitarian development.

He first arrived in Belo Horizonte on December 20th, 1959, and from the first moment, he was immediately enchanted by the city which reminded him of his hometown because of its extremely similar, hilly landscape.

“I am a lot more Brazilian than you, despite the fact that you were born here, I chose to live here.” he would often say when people asked him about his origin.

He got his degree in Medicine from the Medical School of UFMG then went on to specialize in North America for 12 years during which time he received his licenses as a General Surgeon, Vascular and Cardiovascular Surgeon, as well as Thoracic Surgeon working for institutions such as Cleveland Clinic, Henry Ford Hospital, Mount Sinai School of Medicine and Harvard University-Boston Children’s Hospital.

He married Heloisa Angélica Corrêa in 1969. His daughter Erika, cardiologist, and his son Ektor, cardiac surgeon, were both born in Henry Ford Hospital in Detroit while he was a medical resident.

When he returned to Brazil, he taught surgical techniques at the Federal University of Minas Gerais (UFMG) Medical School from 1977 to 2009.

His international professional experience not only gave him knowledge and technical abilities but also enabled him to get involved with biological tissue research with numerous studies and many registered patents in Brazil and throughout the world, in which we highlight the porcine aortic valve (Bioprótese Cardíaca Porcina Biocor), made in Belo Horizonte, Minas Gerais, Brazil. It has been clinically used since 1980, furthermore, since 1997 it is the only product or replaceable valve that can be human implanted, manufactured outside of the United States of America, as well as used in the United States, and approved by the Food and Drug Administration (FDA).

The author of many articles, in his surgical specialization, published in Brazil and abroad, he was also a permanent member of national and international associations.

The dream of the Biocor Institute first opened in 1985 was only possible thanks to his entrepreneurial and innovative profile along with the unconditional support of his beloved wife, Heloisa.

Initially focused on cardiology and cardiac surgery, it grew and gathered different medical specialties necessary to treat patients as a whole with high complexity pathologies.

Mario Vrandecic had always given incentives to continuous education and had provided a good environment for continuous scientific advancement, educating many specialists in his area, who today are recognized and successful professionals working in many different parts of Brazil and abroad.

Alongside their father, Erika and Ektor grew up participating in and contributing to the development of the Biocor Industry and later the Biocor Institute.

As a creator and an entrepreneur, in establishing the Biocor Institute, General Hospital of high complexity, located in Nova Lima, Minas Gerais he stood out by fostering an integrated, informatized and result management system with a focus on human care and continuous improvement, combined with social and environmental responsibility practices. It was the first hospital in Latin America to be certified by ISO 9002 in 1997.

Always present in the institution’s routine, he would fill the hallways of Biocor with vitality and energy, greeting everyone around him with a display of love to his profession and life. Dr. Mario would visit every in-patient in the institution daily, including Sundays and holidays, more than once a day to ensure they had everything they needed.

Acting in society as an engine of change, risk, and people manager, he was always a man ahead of his time. He made the institution a center of technical-scientific and social reference, having the motto “LIFE AS A GREATER VALUE.”

